# Prognostic factors on surgically and non-surgically treated oral squamous cell carcinoma: Advances in survival in fifteen years of follow up

**DOI:** 10.4317/jced.57477

**Published:** 2021-03-01

**Authors:** Paulo-Goberlânio-de Barros Silva, José-Vitor-Mota Lemos, Marcela-Maria-Fontes Borges, Talita-Jordânia-Rocha do Rêgo, Thinali-Sousa Dantas, Carlos-Heli-Bezerra Leite, Marcos-Venício-Alves Lima, Maria-do Perpétuo-Socorro-Saldanha Cunha, Fabrício-Bitu Sousa

**Affiliations:** 1 PhD, Department of Dentistry, Unichristus, Fortaleza, Ceará, Brazil; 2 DDS, Department of Dentistry, Unichristus, Fortaleza, Ceará, Brazil; 3 MSc, Department of Dentistry, Unichristus, Fortaleza, Ceará, Brazil; 4 PhD, Department of Oncology Research, Hospital Haroldo Juaçaba, Fortaleza, Ceará, Brazil

## Abstract

**Background:**

Retrospectively to evaluate the influence of radiochemotherapy (RCT) in the treatment of surgically and non-surgically treated Oral Squamous Cell Carcinoma (OSCC).

**Material and Methods:**

We analysed 934 patients treated in Hospital Haroldo Juaçaba (2000-2014; 15 years of study) by extraction of data type of cancer, localization of tumour, sex, age, race, education level, risk factors (smoking and alcohol use), year of diagnosis, TNM stage, therapeutic approach, health system used (public or private) and overall survival (OS). Surgically and non-surgically treated OSCC were compared by chi-square and Fisher’s exact tests, and their prognostic factors were analysed by log-rank Mantel-Cox plus Cox regression tests (SPSS 20.0, *p*<0.05).

**Results:**

Non-surgically treated OSCC patients had a lower OS than surgically treated OSCC patients (*p*<0.001), but an increase in OS was shown in both groups. Although the 2010-2014 period (*p*=0.003), education level (*p*=0.032), tongue/mouth floor/palate localization (*p*=0.023) and TNM stage (*p*<0.05) were important in non-surgically treated OSCC OS, the major prognostic factors were node metastasis (*p*=0.003) and non-use of RCT (*p*=0.039) (multivariate analysis). In surgically treated OSCC patients, higher OS was shown in the 2010-2014 period (*p*<0.001), females (*p*=0.012), non-drinkers (*p*=0.011), non-smokers (*p*=0.009) and those with lower TNM stage (*p*<0.05), but the major prognostic factor was the 2010-2014 period (*p*=0.004) (multivariate analysis), which was directly associated with an increase in RCT indication (*p*<0.001).

**Conclusions:**

The increase in RCT improved the OS in this large cohort of surgically and non-surgically treated OSCC patients.

** Key words:**Mouth neoplasms, neck, radiotherapy, drug therapy, combination.

## Introduction

Oral cancer is the ninth most prevalent malignant cancer in men and the 15th most prevalent malignant cancer in women. Accounting for the majority of oral cancers, oral squamous cell carcinoma (OSCC) has high incidence and mortality rates, which imposes the need for continuous research on the best therapeutic options (1). OSCC treatment is directly associated with tumour stage. Smaller tumours are treated by radical surgery alone, and larger tumours are treated by a combination of radical surgery plus radiotherapy (RT) or radiochemotherapy (RCT) (2).

Since chemotherapy (CT) does not show significant clinical benefit in OSCC treatment and RT alone has limitations in the treatment of locally advanced tumours (3,4), the combination of RT plus CT is highly beneficial in head and neck tumours (5). The biological basis of RCT benefits from an increase in genetic instability due to CT, raising the efficacy of RT (6).

The first clinical trial of RCT in head and neck cancer treatment was conducted in 1986, showing good control of locally advanced tumours compared to that with RT alone (7). Since then, some studies have accumulated good scientific evidence of RCT benefits in the control of late-stage head and neck cancer and locally advanced oral cancer (8,9).

RCTs can be combined with radical surgery, which is called trimodal therapy (surgery plus RCT). Trimodal therapy reduces recurrence rates and prolongs overall survival in OSCC patients (10). Since RCT use is increasing in surgically and non-surgically treated OSCC, the objective of this study was to evaluate the influence of increased RCT use in the treatment of surgically and non-surgically treated OSCC in a large Brazilian cohort.

## Material and Methods

-Study design and data collection

This is a quantitative retrospective study in which we evaluated a convenience sample of 934 OSCC patients. All patients were diagnosed and treated in Hospital Haroldo Juaçaba (Instituto do Câncer do Ceará) between January 1, 2000, and December 31, 2014 (15 years of study).

The data were extracted from the Register of Cancer Sector. The database has information about type of cancer, localization of tumour, sex, age, race, education level, conventional risk factors (smoking and alcohol use), year of diagnosis, TNM stage, therapeutic approach, health system used (public or private), and dates for start of the treatment, last follow-up and death. We included only OSCC patients with completed treatment, and we divided the patients into surgically and non-surgically treated groups. From then on, we obtained all usable information. Patients without a treatment protocol and patients who underwent interrupted/modified treatment were excluded.

Survival (months) was calculated by the difference between the date of the start of treatment and the date of death (11).

-Radiation Therapy and Concomitant Systemic Therapy

External beam with linear accelerators was the main radiation modality and before 2003 only conventional-2D was available in our centre. At that time, patients were immobilized with thermoplastic masks and bite blocks. Cervical mass and/or scars were identified using radiopaque markers. Gross disease received 70 Gy, high risk drainage was treated with 60 – 66 Gy with parallel and opposite fields and the low neck with 50 Gy using a direct appositional field (source-surface-distance technique) with all regions receiving 2 Gy per fraction and 5 days per week. After conformal-3D technique integrates our routine in 2002 the same principles were generally followed in relation to dose and fractionation.

Intensity Modulated Radiation Therapy (IMRT) became available to our department in 2012. The most common protocol used Simultaneous Modulated Accelerated Radiation Therapy (SMART) / Simultaneous Integrated Boost – SIB, in 33 – 35 fractions in primary radiotherapy and up to 30 fractions in adjuvant intent. Dose was 70 Gy to gross disease, 66 Gy to zones with nodal extracapsular extension and/or positive margins and 54-56 Gy to low risk volumes (ensuring a minimal daily dose of 1.6 Gy per fraction).

When indicated, cisplatin (100 mg/m2) on days 1, 21 and 43 (preferred) or (80 mg/m2) on days 1, 21 and 43 or a conversion to a weekly scheme dose of 30mg / m2 is used (patients with poorer performance) is used in association to RT. When abnormal creatinine clearance is reduced (< 60 ml/min), carboplatin is used (AUC 1.5 – 2) replacing cisplatin. Few cases were treated with a specific protocol using weekly carboplatin (AUC 1.5 – 2) plus paclitaxel (50 mg / m2) and few elderly patients were treated with concomitant target therapy (in instead of CT) with cetuximab (400 mg / m2) given 1 week before and weekly during radiation.

-Statistical analysis

All data were analysed by SPSS software (Statistical Package for Social Sciences) for Windows version 20.0 with a confidence level of 95% (*P* < 0.05).

We calculated the incidence of new cases of surgically and non-surgically treated OSCC, and we expressed the data such as absolute and percentual frequencies. Chi-square tests and Fisher’s exact tests were used to compare all variables. Therefore, the Kaplan-Meier method was used to determine overall survival (OS), which was analysed by the log-rank Mantel Cox method plus the Cox regression model of survival.

-Ethical Correlations

This study conformed to ethical principles and was approved by the ethics committee of Hospital Haroldo Juaçaba under protocol 2,191,839.

## Results

-Clinical prognostic factors in surgically and non-surgically treated OSCC patients over 15 years of follow-up

We evaluated 934 OSCC patients who underwent the treatment approach; 466 OSCC patients were surgically treated, and 468 were non-surgically treated. Most patients were male (n=660, 70.7%), were over 60 years old (n=508, 54.4%), brown (n=583, 63.9%) completed grade school (n=422, 57.3%) and were treated by the public health system (n=492, 85.9%). These characteristics did not differ between treatment approaches (Table 1,  Table 1 cont.).

**Table 1:**
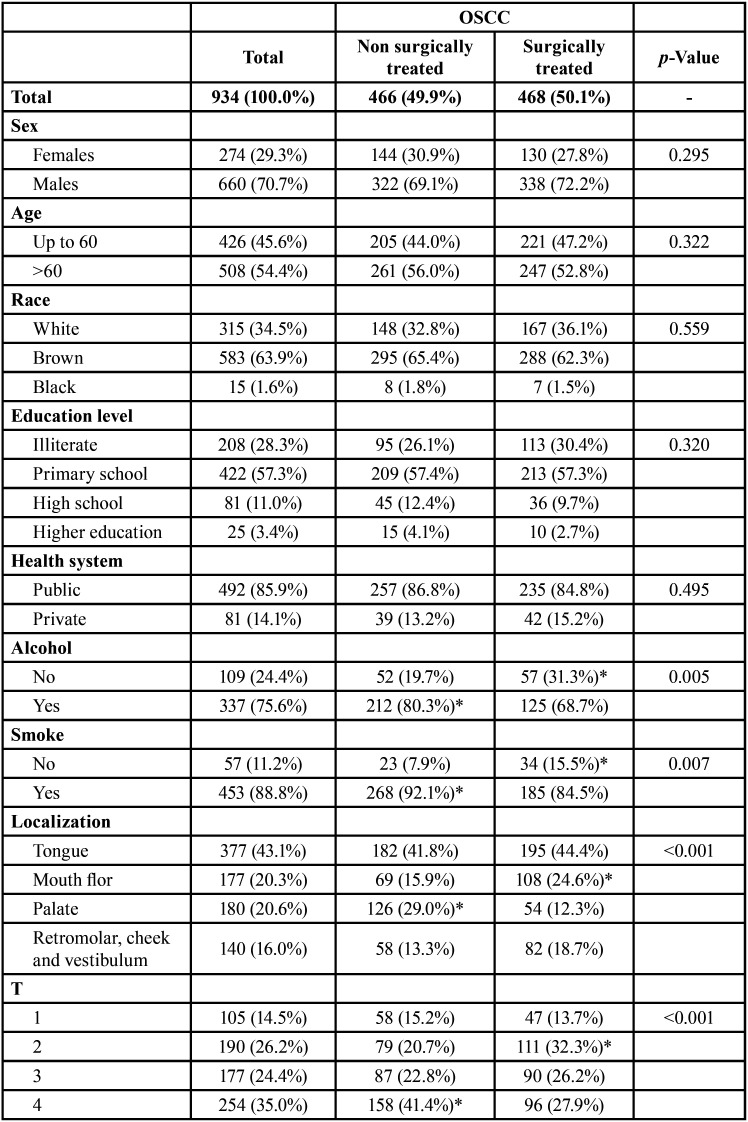
Influence of sociodemographic and clinic characteristics in surgically and non-surgically treated OSCC profile in Hospital Haroldo Juaçaba (Ceará Cancer Institute) during the period from January 1, 2000 to December 31, 2014.

**Table 1 cont.**
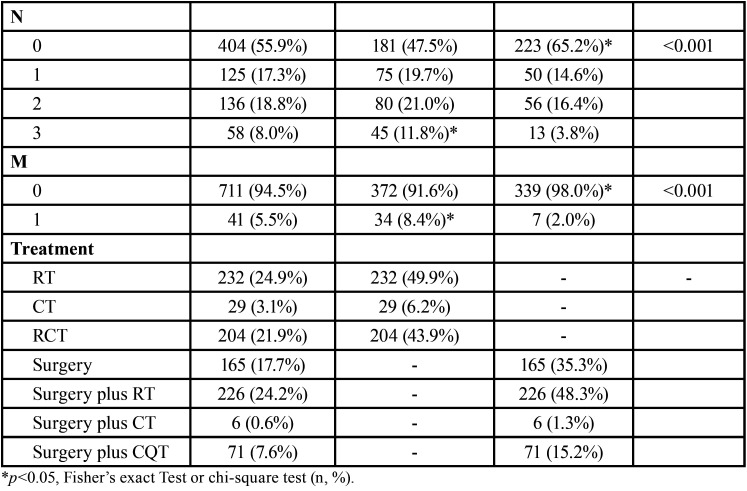
Influence of sociodemographic and clinic characteristics in surgically and non-surgically treated OSCC profile in Hospital Haroldo Juaçaba (Ceará Cancer Institute) during the period from January 1, 2000 to December 31, 2014.

Alcohol consumption (n=337, 75.6%) and smoking (n=453, 88.8%) were noted in most patients and were significantly associated with non-surgical treatments (*p*=0.005 and *p*=0.007, respectively) (Table 1,  Table 1 cont.).

The tongue was the most prevalent anatomical site (n=377, 43.1%), and the clinical stage was predominantly T4 (n=254, 35.0%), N0 (n=404, 55.9%) and M0 (n=711, 94.5%). T4 (*p*<0.001), N3 (*p*<0.001) and M1 (*p*<0.001) tumours were significantly more frequently treated by non-surgical approaches. The most prevalent surgical approaches were surgery plus RT (n=225, 24.2%) or surgery alone (n=165, 17.7%), and the most prevalent non-surgical approaches were RT (n=232, 24.9%) and RCT (n=204, 21.9%) (Table 1,  Table 1 cont.).

The average annual number of OSCC cases was 62/year. Both surgically and non-surgically treated OSCC showed increasing incidence between 2000 and 2014 (*p*<0.001). The non-surgical cases had an annual growth rate of 37%, and the surgical cases had a growth rate of 30%.

-Clinical prognostic factors and therapeutic approaches influenced the OS of surgically and non-surgically treated OSCC patients over 15 years of follow-up

The 15-year OS of surgically treated patients was significantly higher than that of non-surgically treated patients (*p*<0.001). Both surgically (*p*=0.003) and non-surgically treated OSCC patients (*p*<0.001) showed higher OS when diagnosed between 2010 and 2014 than when diagnosed during other periods. Sex was not a prognostic factor associated with OS in non-surgically treated OSCC (*p*=0.307), but women showed high OS in the surgically treated OSCC group (*p*=0.012) (Table 2,  Table 2 cont.).

**Table 2:**
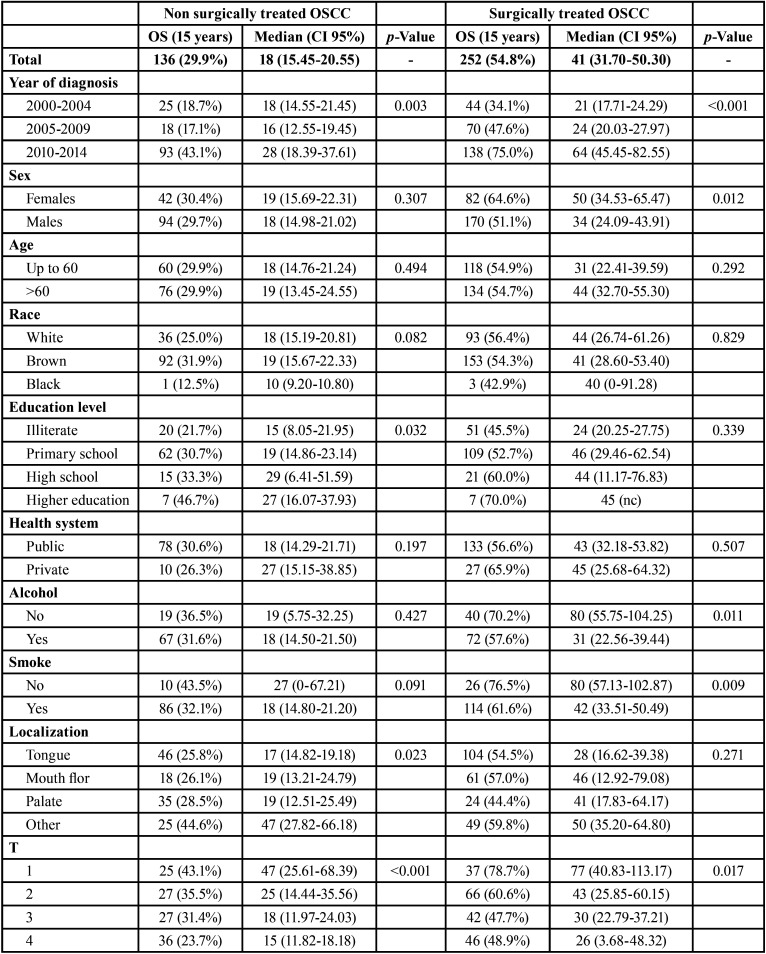
Influence of sociodemographic and clinic-therapeutics characteristics in OS of surgically and non-surgically treated OSCC in Hospital Haroldo Juaçaba (Ceará Cancer Institute) during the period from January 1, 2000 to December 31, 2014.

**Table 2 cont.**
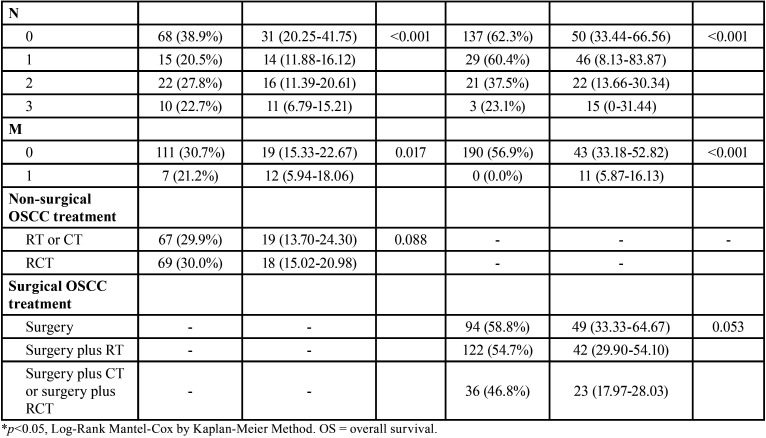
Influence of sociodemographic and clinic-therapeutics characteristics in OS of surgically and non-surgically treated OSCC in Hospital Haroldo Juaçaba (Ceará Cancer Institute) during the period from January 1, 2000 to December 31, 2014.

Age, race and health system were not prognostic factors in OSCC (*p*>0.05). However, although schooling was not associated with the OS of surgically treated patients (*p*=0.339), in non-surgically treated patients, it was directly associated with the best OS (*p*=0.032). Alcohol consumption (*p*=0.427) and smoking (*p*=0.091) were not associated with prognosis in non-surgically treated OSCC, but in surgically treated OSCC, lower OS was shown in patients with these two risk factors (*p*=0.011 and *p*=0.009, respectively) (Table 2,  Table 2 cont.).

Tongue, floor of mouth and palate tumours showed lower OS than retromolar, cheek and vestibulum of the mouth tumours in non-surgically treated OSCC (*p*=0.023). In surgically treated patients, the anatomical site was not a significant prognostic factor (*p*=0.271). High clinical TNM stage was significantly associated with poor OS in surgically and non-surgically treated OSCC patients (Table 2,  Table 2 cont.).

In non-surgically treated OSCC, RT plus CT showed only slightly lower OS than RT alone (*p*=0.088). In surgically treated OSCC, surgery alone had only a slightly better OS than surgery plus RT, plus CT or plus RCT (*p*=0.053) (Table 2,  Table 2 cont.).

Multivariate analysis showed that Year of diagnosis (*p*=0.119), Sex (*p*=0.600), Age (*p*=0.302), Race (*p*=0.567), Education level (*p*=0.802), Alcohol (*p*=0.930), Smoke (*p*=0.924), Health system (*p*=0.569), Localization (*p*=0.681), T (*p*=0.678) and M (*p*=0.457) did not influenced OS in non-surgically treated OSCC and determinant factors for OS in non-surgically treated OSCC were clinic stage N (*p*=0.003, HR = 3.94, CI95% = 1.60-9.73) and RCT non-surgical protocols (*p*=0.039, HR = 2.16, CI95% = 1.05-4.46). In surgically treated OSCC, Sex (*p*=0.568), Age (*p*=0.997), Race (*p*=0.362), Education level (*p*=0.837), Alcohol (*p*=0.114), Smoke (*p*=0.617), Health system (*p*=0.274), Localization (*p*=0.575), T (*p*=0.979), N (*p*=0.558), M (*p*=1.000), Treatment (*p*=0.354) did not influenced OS, but diagnosis between 2010-2014 (*p*=0.004, HR = 4.83, CI95% = 4.83-13.99) was the determinant factor for a better OS.

-An increase in RCT protocols directly or indirectly impaired the OS of surgically and non-surgically treated OSCC patients

In non-surgically treated OSCC patients, the RCT was most used in patients diagnosed between 2010-2014 (*p*=0.004), males (*p*<0.001), <60 years old (*p*<0.001), alcohol consumers (*p*=0.018), and those with N+ stages (*p*<0.001). In surgically treated OSCC, RCT was most commonly used in patients diagnosed between 2010-2014 (*p*<0.001), males (*p*=0.005), patients <60 years old (*p*<0.001), alcohol consumers (*p*=0.029), smokers (*p*=0.020, T2-4 (*p*=0.025) and those with N+ stages (*p*<0.001) (Table 3).

**Table 3:**
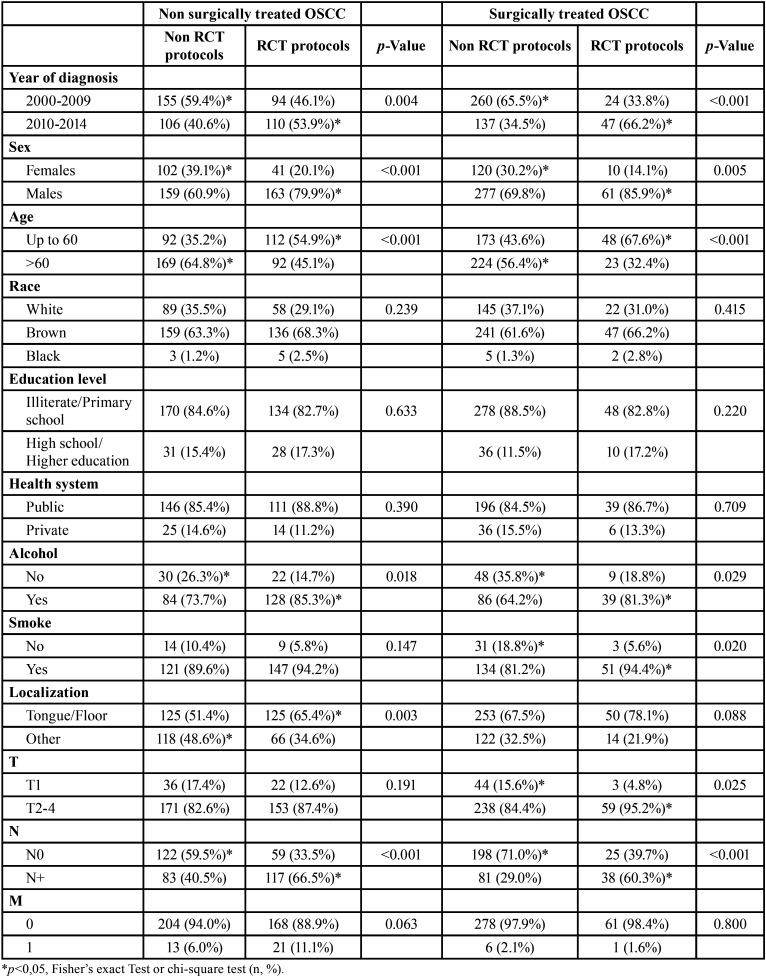
Influence of sociodemographic and clinic characteristics in indication of RCT protocols on surgically and non-surgically treated OSCC profile in Hospital Haroldo Juaçaba (Ceará Cancer Institute) during the period from January 1, 2000 to December 31, 2014.

The non-surgically treated OSCC patients diagnosed between 2010-2014 were mostly brown (*p*<0.001) and treated by the public health system (*p*=0.038) and the RCT protocol (*p*=0.004). The surgically treated OSCC patients diagnosed between 2010 and 2014 were mostly brown (*p*=0.005), alcohol consumers (*p*=0.045) and treated by RCT protocols (*p*<0.001) (Table 4).

**Table 4:**
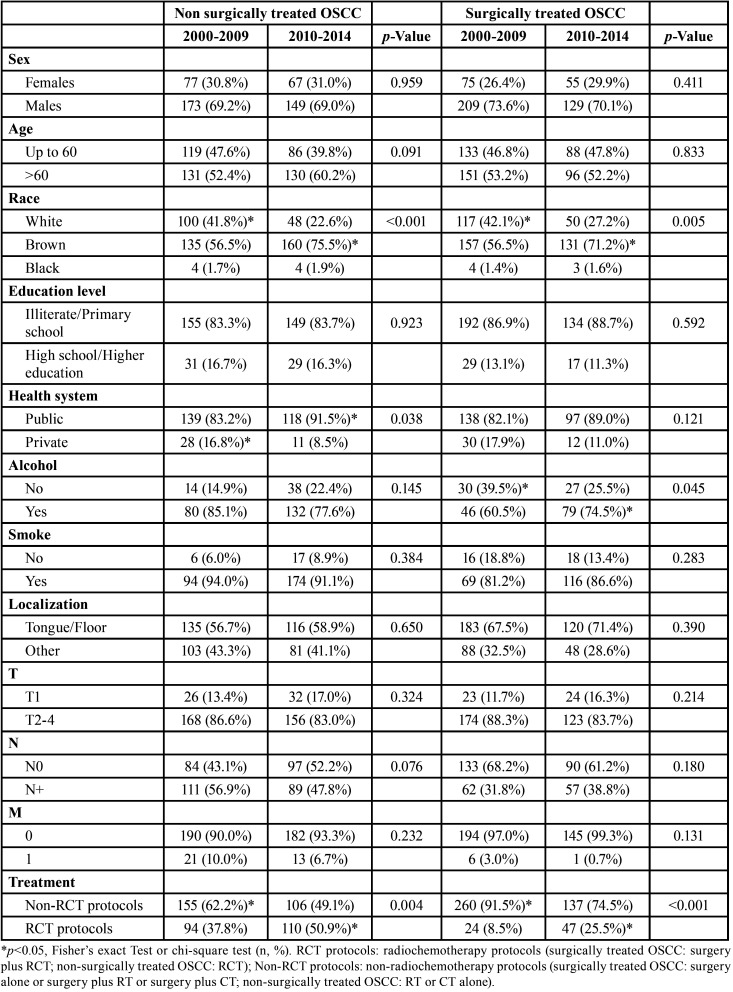
Influence of sociodemographic and clinic characteristics in period of treatment on surgically and non-surgically treated OSCC profile in Hospital Haroldo Juaçaba (Ceará Cancer Institute) during the period from January 1, 2000 to December 31, 2014.

## Discussion

This research showed a trend towards an increase in the incidence of non-surgically treated OSCC in a Brazilian high complexity oncology centre. The number of new cases by year of non-surgically treated OSCC was 7% higher than that of surgically treated OSCC. These results were contradictory to US studies that show an increase in OSCC incidence in a similar period (2004 to 2013) but a slowdown in the growth of non-surgically treated OSCC rates (12). Studies with smaller cohorts in Brazil showed a similar tendency of increase in non-surgically treated OSCC because in Brazil, the late diagnosis in is primarily responsible for the increase observed in the public health system in the 2010-2014 period (13,14).

We did not show differences in indication of non-surgical treatment between sex, age, race, schooling and health system. These results vary from sample to sample, and normally, men were non-surgically treated due to their higher clinical stages and young age and their better tolerance of RT and CT side effects (15). Non-surgical protocols were too frequently used palliatively in patients with low income and low education (12).

Although the late stages of OSCC with non-surgical protocol indications have a social component (11), we did not observe differences between schooling levels and non-surgical protocol indications. However, smoking history and alcohol consumption are most prevalent in low-income people, impairing the high incidence of late stages of OSCC (16).

Although studies have shown a higher indication of non-surgical protocols in tongue OSCC (15), in our sample, tongue OSCC was equally indicated for surgical and non-surgical treatment protocols (12). Conversely, palate OSCC has a high indication of non-surgical treatment protocols due to difficulty in surgically addressing palate tumours and due their response to RT (17,18).

Classically, clinical stage is directly associated with an increase in the indication of non-surgical treatment for OSCC. Stages III and IV are strongly suggested for RT and RCT (19). In non-surgical cases of our cohort, we showed a discrete, high indication of RT and a significant indication trend of RCT in the 2010-2014 period. OSCC treated with surgery plus RT or surgery alone was the most prevalent treatment protocol used, and RCT was most commonly used in the 2010-2014 period, suggesting an increase in the late diagnosis of OSCC.

In surgically treated OSCC, the OS was 41 months, tripling in the 2010-2014 period. In non-surgically treated OSCC, the OS was 18 months, with an increase of 50% in OS in the 2010-2014 period. Surgical treatment of OSCC is classically more prevalent than non-surgically treated OSCC , and despite the increase in incidence of non-surgically treated OSCC in the 15-year follow-up period, the OSCC modification of the therapeutic profile (increase in RCT protocol treatment) increased the 15-year OS of surgically and non-surgically treated OSCC patients. Similar results were previously described, especially in low stage tumours (20,21).

In the univariate analysis, poor schooling and tongue/palate/floor of the mouth showed lower OS, but the clinical stage was the major risk factor for poor survival. In the multivariate analysis, lymph node metastasis and non-RCT protocol treatment were the major risk factors for poor OSCC survival. In a previous extensive literature review, RCT treatment protocols showed a better prognosis for head and neck tumours, and in India, RCT-treated head and neck cancers showed the best therapeutic response (19,22). In Japan, stage III and IV RCT-treated OSCC showed a complete response when RCT protocols were used, and in German neoadjuvant RCT before surgery, a high 10-year OS of 66.7% was observed despite most deaths of OSCC occurring within the first two years after treatment (23-25).

In a clinical trial comparing RCT (cisplatin plus cetuximab) in head and neck tumours with a late stage and versus head and neck tumours with early stages to those treated by surgery plus RT, the non-surgical protocols resulted in similar OS between the groups (26). Compared to RT, RCT increased the response rate in stage III and IV mouth, hypopharynx, laryngopharynx and hypopharynx tumours, showing improved 5-year OS; nodal involvement was, similar to our study, the major risk factor in non-surgically treated OSCC (27).

RCTs have a strong response ratio (80%) in head and neck tumours (28). However, despite the excitement of RCT use in head and neck tumours, clinicians must be cautious . In our samples, RCT was most commonly used in men and young patients, in both surgically and non-surgically treated cases; cisplatin or carboplatin plus 35-60 Gy of RT are the most common RCT protocols and are associated with some side effects (24).

The main RCT-related adverse effects are osteoradionecrosis (11-36%) and oropharyngeal mucositis, which makes eating difficult and led to significant weight loss (21,29). Additionally, the presence of comorbidities, which are most common not in young individuals but in older individuals, increases the incidence of side effects (15,30). Therefore, it is necessary that patients be in good health to start RCT. Thus, there are many considerations regarding the choice of RCT as a surgical or non-surgical-associated therapeutic modality in patients with oral OSCC, especially in elderly patients.

Our study has the common limitations of retrospective studies. Even though our samples are from a large oncology reference centre in Brazil, we have data loss and fill bias, and our records summarily grouped the mouth RCT protocols (cisplatin, 5-fluorouracil, carboplatin, tirapazamine or cetuximab and others plus some RCT fractionations). Nevertheless, the OS increase shows a great trend of non-surgical treatment of the head. Both surgically and non-surgically treated OSCC benefited from RCT treatment protocols, but protocol-specific guided studies must be performed to show the best RCT protocols for the head and neck to increase the OS of OSCC.
